# Design and experimental validation of a smart alkaline electrolyzer-based green hydrogen generation and storage pilot system

**DOI:** 10.1038/s41598-026-64755-7

**Published:** 2026-08-07

**Authors:** Helmy M. El Zoghby, Mahmoud M. Elmesalawy, Ibrahim Shindi

**Affiliations:** 1 Department of Electrical Power and Machines Engineering, Helwan University, Cairo, 11792 Egypt; 2 Department of Electronics and Communication Engineering, Helwan University, Cairo, 11792 Egypt

**Keywords:** Green hydrogen, Alkaline electrolyzer, Renewable energy, Smart control, PLC automation, Hydrogen storage, Energy science and technology, Engineering, Environmental sciences

## Abstract

There are many technologies for storing electrical energy, some of these are environmentally friendly and some cause environmental pollution, which leads to global warming. Therefore, designing and using an environmentally friendly way to store electrical energy is one of the most important priorities in the current era. The way to store electrical energy in the form of hydrogen is one of the most promising ways to store electrical energy in all countries. This article deals with a comprehensive practical study of the design and implementation of 0.5 kW green hydrogen production and storage system powered by solar energy. The stored hydrogen is used for feeding AC and DC loads through fuel cell. In this research the water Electrolyzer is completely designed and implemented. Supervisory control and data acquisition (SCADA) system is used to monitor and control generation, storage and usage stages. The most important contribution of this research is the step-by-step precise design and implementation of water Electrolyzer, considering the necessary precautions during the design process, conducting numerous tests on this Electrolyzer, and arriving at recommendations that will be useful in the design of water analyzers in the future. The designed Electrolyzer optimal input voltage range 2.4 to 2.6 V DC, Electrolyzer input current range 210 to 250 A, Electrolyzer input power range 500 to 700 W, Hydrogen peak output rate 4.5 L/min of pure H_2_ that equivalent to 6.75 L/min of HHO mixture. Theoretical energy conversion efficiency ~ 67% but practical efficiency is ~ 62 to 66%, based on gas volume and input energy.

## Introduction

Hydrogen is considered one of the fundamental elements in the universe, carrying energy. Hydrogen is the most abundant element in the universe, accounting for approximately 75% of its baryonic mass (and about 90% of its atoms. It is found in water, the bodies of animals, plants, humans, and birds, as well as in petroleum and its derivatives, and in coal^1^. Electrolysis is a modern method for obtaining hydrogen from water. The Electrolyzer works by breaking down water into hydrogen and oxygen using electrical energy, which in the modern era is often obtained from renewable energy sources such as the sun and wind^2^. Once hydrogen is produced, it must be stored, transported, and reused safely. Therefore, this article discusses the optimal design of a water Electrolyzer, the necessary precautions, and the safety measures to be followed when producing, transporting, storing, and reusing hydrogen.

Electrolyzers are categorized according to their construction and operational features. Alkaline Electrolyzers (AEL), solid oxide Electrolyzers (SOE), and proton exchange membrane (PEM) Electrolyzers are the three primary types^3^. The maturity and affordability of alkaline Electrolyzers have led to their widespread adoption. It functions at moderate temperatures between 60 °C and 80 °C and use a liquid electrolyte, usually potassium hydroxide (KOH)^4^. Compact design and greater operating pressures are made possible by PEM Electrolyzers, which use a solid polymer membrane as the electrolyte. The optimal temperature range for these Electrolyzers is between 50 °C and 70 °C^5^. Lastly, SOE Electrolyzers use steam as the feedstock and run at much higher temperatures, between 600 °C and 1000 °C. Increased efficiency is possible with this high-temperature operation, although material stability issues arise^6^. Electrolyzer performance is determined by several important characteristics. Depending on the system’s kind and efficiency, the applied voltage usually falls between 1.8 and 2.5 volts per cell^7^. In^8^ a description of the design and performance evaluation of a novel 1 kW-class hydrogen production/power generation system is done. A crucial factor influencing the rates of hydrogen production, current density typically ranges from 0.2 to 2 amperes per square centimeter. Higher current densities can have a detrimental effect on durability and efficiency even when they increase production rates. Another crucial element is operation temperature; AEL systems run at lower temperatures than SOE systems, which need high heat to function at their best. These systems’ overall efficiency ranges from 60% to 80%, and research is still being done to increase this figure^9^.

Electrolyzer manufacture is a complex process. Electrode manufacture is a crucial stage that necessitates the use of robust materials, such as nickel, platinum, or iridium, which are frequently coated to improve catalytic performance^10^. While SOE Electrolyzers use ceramic materials for their electrolytes, PEM Electrolyzers depend on the creation of proton-conductive membranes^11^. After each part is ready, it is placed together into stacks with cells connected in series to produce the desired result. To guarantee that the stacks can endure operational pressures and temperatures while retaining durability over time, the last steps entail sealing and enclosing the stacks^12^. After hydrogen is created, it must be stored effectively to allow for its transportation and usage in a variety of applications. The purpose of hydrogen storage tanks is to securely hold hydrogen in either its liquid or gaseous state. The most popular are compressed gas tanks, which hold hydrogen at pressures between 350 and 700 bar^13^. Because of their lightweight construction and comparatively straightforward design, these tanks are frequently utilized in transportation applications. As an alternative, liquid hydrogen is stored in cryogenic tanks at very low temperatures, usually − 253 °C, which offers a higher energy density but necessitates sophisticated insulation to avoid boiling off^14^. Although they typically have smaller storage capacities, metal hydride tanks are another approach in which hydrogen is absorbed into metal alloys for safe storage at lower pressures^15^.

The use and storage technique dictate the requirements of hydrogen storage tanks. For high-pressure compression tanks, which are usually made of carbon fiber composites, material strength is very important^16,17^. For transportable and portable tanks, these materials offer the required strength-to-weight ratio. In contrast, cryogenic tanks are typically constructed from aluminum alloys or stainless steel to provide structural integrity and efficient thermal insulation at low temperatures. The size and technology of the tank determine the storage capacity, which is usually expressed in kilos of hydrogen. Additionally, pressurized tanks need to be resilient to heat stress and wear to endure quick cycles of filling and discharging^18^. There are several clearly defined steps involved in the manufacturing of hydrogen storage tanks. The first step in the process for high-pressure tanks is to choose lightweight, strong materials, including carbon fiber composites^19^. The tank body is created by winding these materials around a core using sophisticated filament winding processes. For cryogenic applications, metallic tanks are molded or welded to exact specifications. To stop hydrogen leaks, a metal or polymer inner liner is incorporated into the tank^20^. To make sure the tanks satisfy safety requirements, extensive quality testing is carried out after assembly, including pressure cycling and burst tests.

The work in this paper stressed that the uniqueness and contribution of this study lie in its actual implementation within a full 0.5 kW pilot-scale green hydrogen generation system rather than only in giving a thorough Electrolyzer design. The system integration strategy, component selection and sizing, operating methodology, experimental validation under actual operating conditions.

The main contributions of the paper are : (1) the development of an integrated automation and monitoring architecture that permits real-time process control, data acquisition, and operational supervision; (2) the full engineering design and experimental validation of a 0.5 kW pilot-scale alkaline Electrolyzer system under realistic operating conditions; (3) a thorough experimental assessment of the system, including electrical performance, hydrogen production rate, and overall system efficiency under various operating conditions; and (4) the conversion of laboratory concepts into a scalable pilot-scale platform that can function as a basis for future hydrogen production systems powered by renewable energy. We have also extended the comparison with previous relevant works to better elucidate the scientific contribution, emphasizing the ways in which the suggested system varies with respect to scale, system architecture, experimental validation, control implementation, and practical operation.

The research and implementation of Electrolyzers and hydrogen storage tanks continue to face obstacles despite notable progress. Cost, scalability, and efficiency at high current densities are obstacles that Electrolyzers must overcome. Like this, storage tanks must strike a compromise between cost, safety, and storage density; both cryogenic and pressurized systems have drawbacks^21^. Materials science developments, such the creation of new catalysts for Electrolyzers and lightweight composites for tanks, show promise in resolving these issues. A route to more effective hydrogen systems may potentially be provided by hybrid storage systems, which combine the advantages of cryogenic and compressed technology^22^.

Alkaline Electrolyzers were selected in this work because they are mature and commercially proven technology, offering high reliability, relatively low capital cost, and long operational lifetime compared with other Electrolyzer technologies. They also employ non-precious metal electrodes and an inexpensive alkaline electrolyte, making them particularly suitable for pilot-scale and cost-sensitive applications^23^.

## The proposed system under investigation

**Fig. 1:**
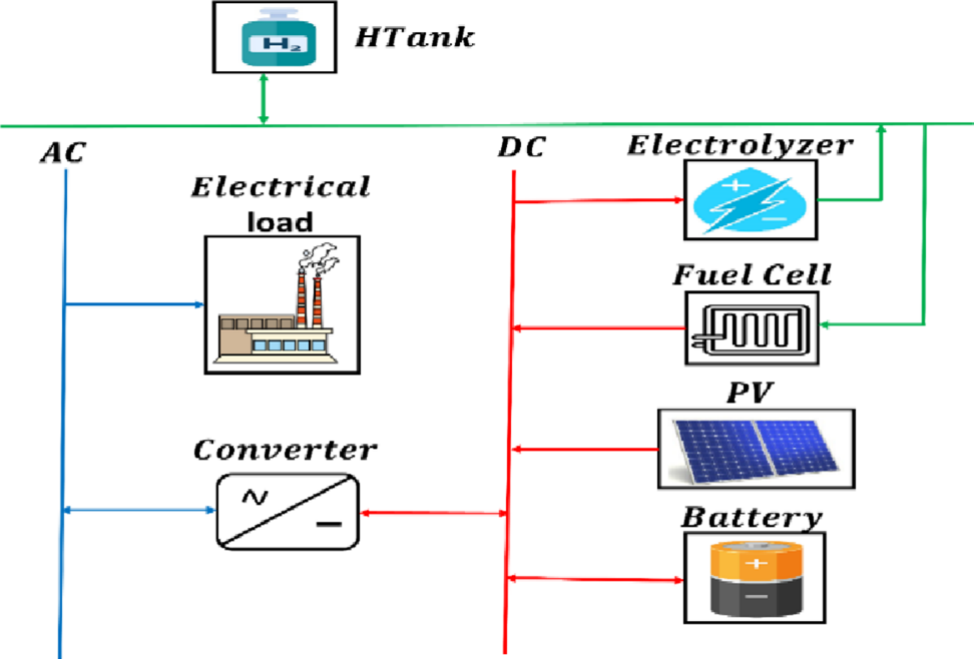
Proposed system under investigation.

**Fig. 2:**
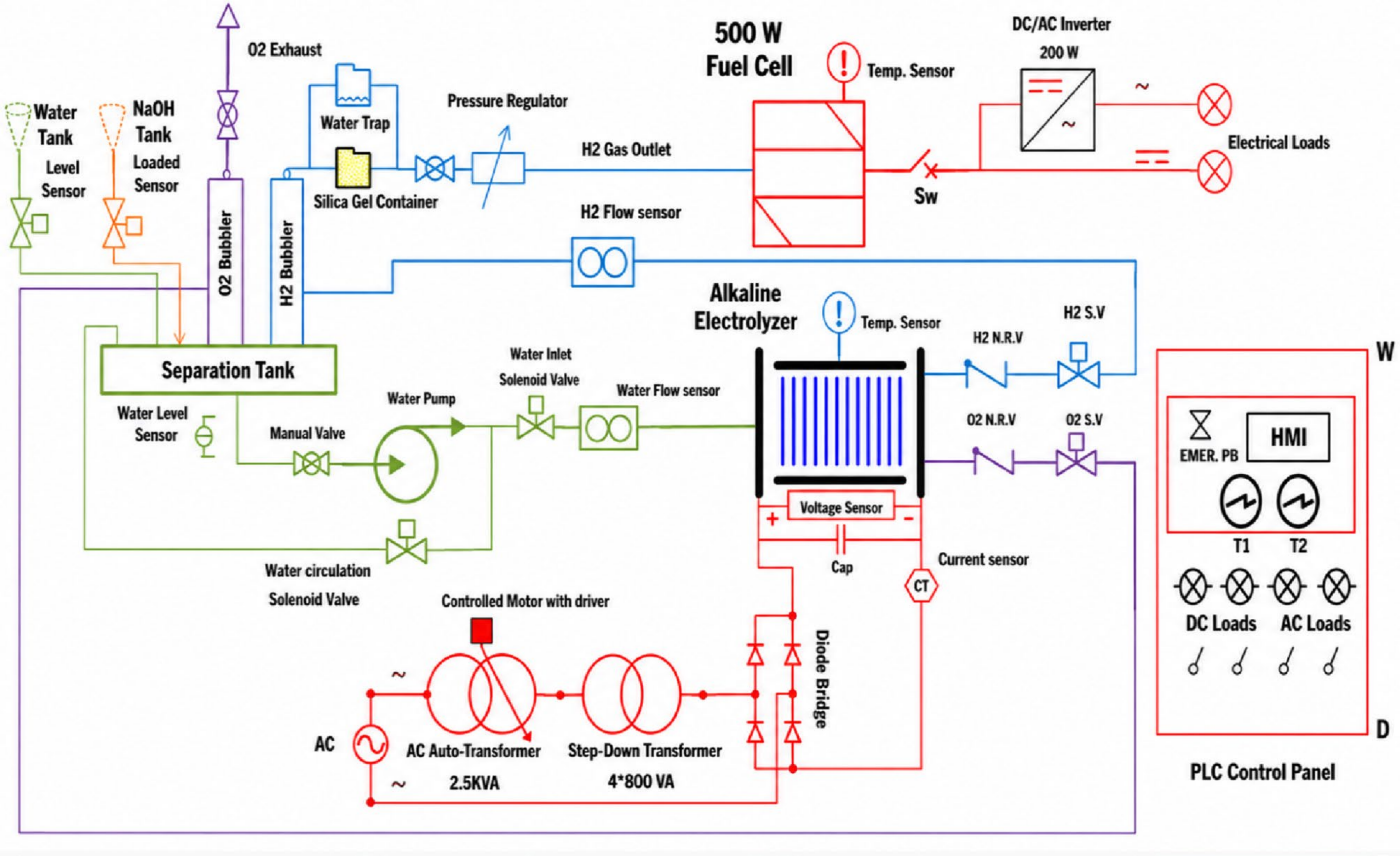
Experimental setup supplied from temporary AC source (grid-based).

**Fig. 3:**
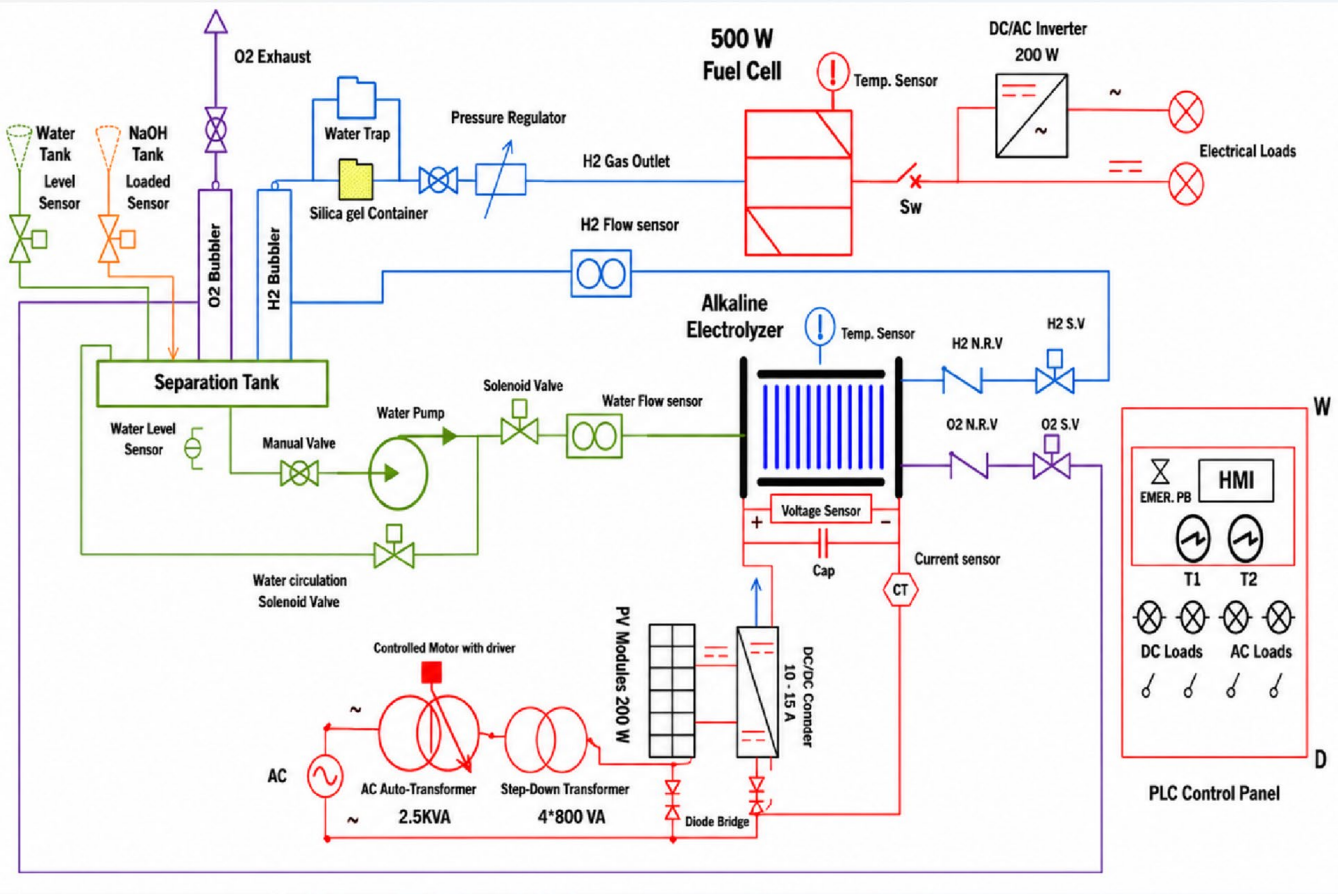
Experimental Setup supplied from permanent PV source (renewable based).

**Fig. 4:**
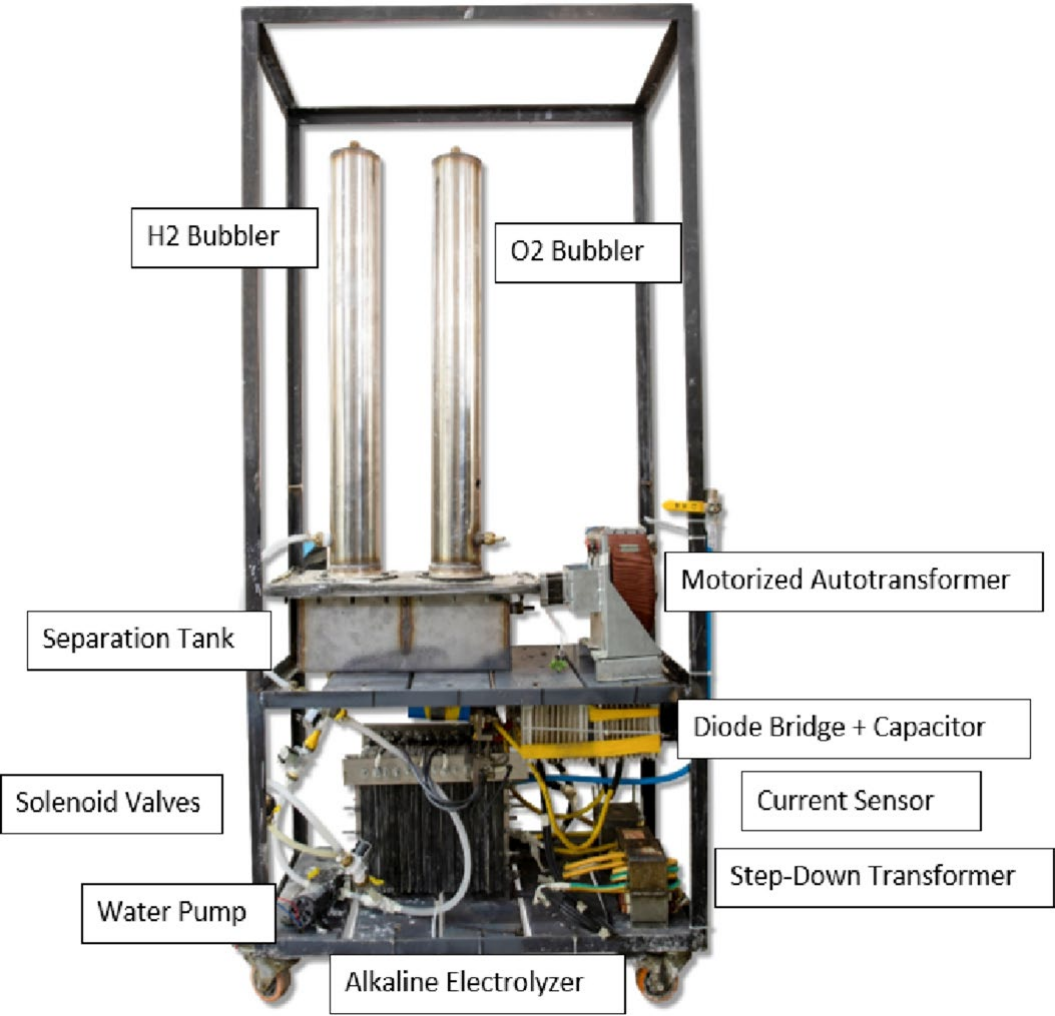
Photograph of the interior structure of the practical prototype.

Solar energy is captured by solar panels (PV) and converted into electrical power. This electricity is transformed to meet the needs of the microgrid after going via a converter. Excess energy is stored in batteries, which act as a ready-to-use backup during periods of low sunlight. A green hydrogen system is employed when the output of renewable energy is insufficient (Fig. 1). The Electrolyzer creates hydrogen gas by splitting water molecules using extra power. This hydrogen is then stored in a secure tank. By transforming the hydrogen, it has stored back into energy when needed, the fuel cell powers the microgrid and the associated load, such as the adjacent cable factory. This combination of storage options, conversion technologies, and renewable energy sources ensures a steady and sustainable energy supply for the island community. As illustrated in Figs. 2 and 3, hydrogen can be produced using a temporary power supply or substituted by renewable energy sources like photovoltaic (Fig. 4).

## Electrolyzer fabrication

**Fig. 5:**
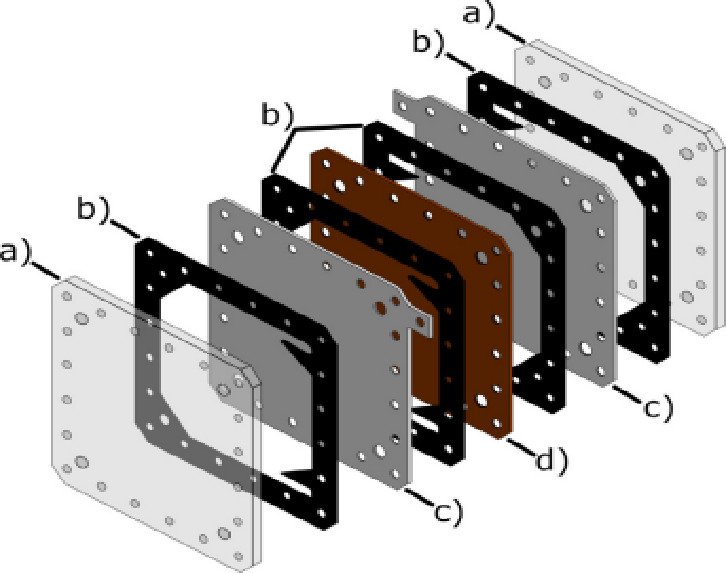
Photograph of the electrolyzer components.

The Electrolyzer is the enclosure where the chemical reactions take place in, therefor it must be completely enclosed so that the gases don’t Leaked from it and affect the quality and the quantity of the out gases, the Electrolyzer is designed under certain aspects which determined under the scale of the system and the purpose of the experiment. The installed Electrolyzer in this work is the Alkaline Electrolyzer which has several advantages over the other varieties of Electrolyzers. Alkaline Electrolyzers are often manufactured using materials that are resistant to corrosion and can endure the extreme conditions of the electrolysis process. The primary materials utilized in alkaline Electrolyzers as illustrated in Fig. 5 are:

- Electrodes: The electrodes in alkaline Electrolyzers are commonly formed of Stainless-Steel Sheets (Anode and Cathode) “Polyester fiber membrane”. These materials are resistant to corrosion and can sustain the extreme conditions of the electrolysis process.
- Membrane: Alkaline Electrolyzers use a liquid NAOH electrolyte.
- Cell components: The cell components of alkaline Electrolyzers, such as cell frames, separators, and pressure vessels, are commonly constructed of materials such as stainless steel, carbon steel, or nickel alloys. These materials are resistant to corrosion and can sustain the extreme conditions of the electrolysis process.

The Electrolyzer active electrode area was determined from the desired power rating using the relationships *\:P=VI* and *\:I=JA*, yielding $\:A=P/\left(VJ\right)$, where J is the current density, A is the area. Therefore, the physical dimensions of the electrolyzer were selected to provide the required active area while satisfying the target operating current density, in accordance with standard electrochemical engineering design methodology^17^.

where (a) represents 2 translucent acrylic plates, (b) is 4 neoprene seals and (c) is 2 stainless steel sheets (anode and cathode) polyester fiber membrane.

**Fig. 6:**
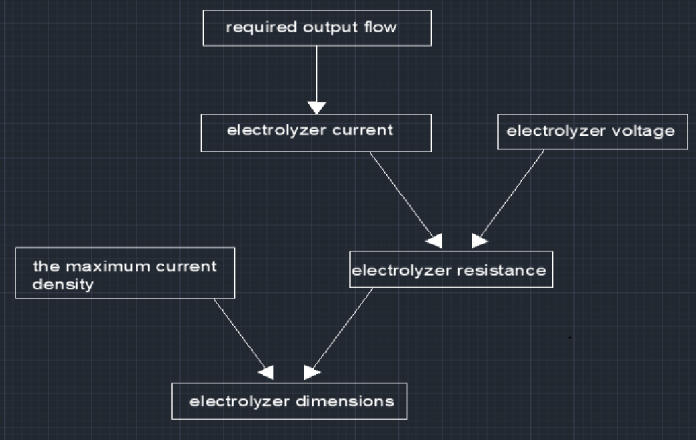
The Electrolyzer cell design requirement.

**Fig. 7:**
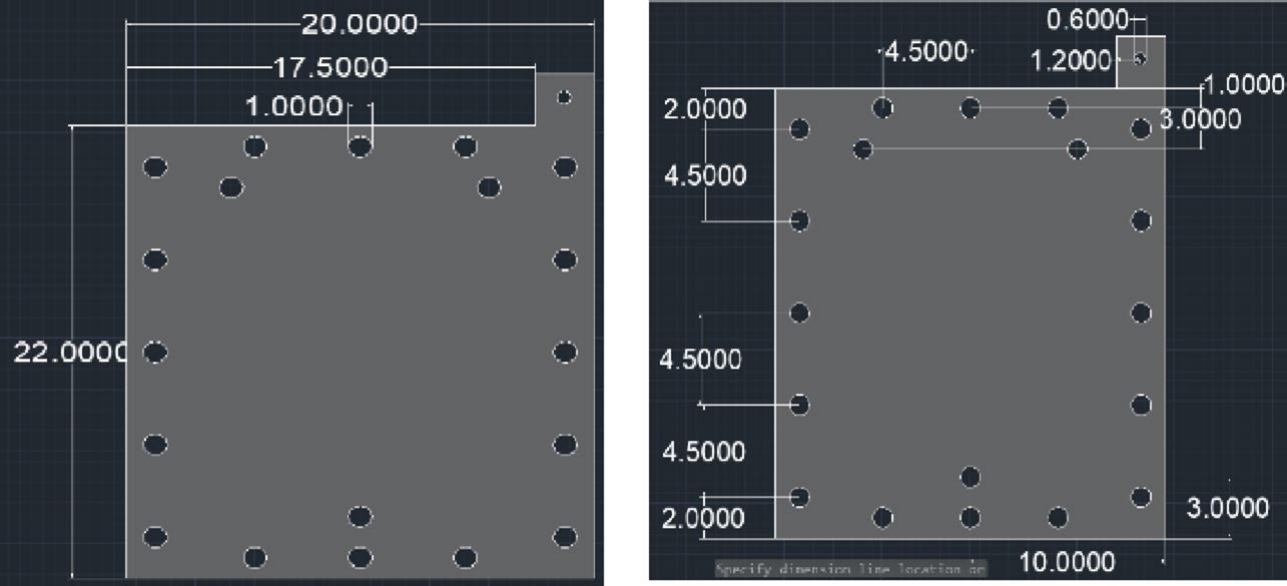
Dimensions of anode and cathode plates.

Figure 6 depicts the Electrolyzer cell design need, The proposed Electrolyzer dimensions of anode and cathode plates: The dimensions of anode and cathode plates as indicated in Fig. 7 are selected as 200 mm x 220 mm width and height respectively. The diameter of each clamping hole is specified as 10 mm. The outside diameter of electrolyte supply tube is specified as 10 mm. The cathode side base plate is precisely identical to the anode side base plate. The thickness of the electrode is 0.6 mm, electrode space of the Electrolyzer is specified as 10 mm width.

**Fig. 8:**
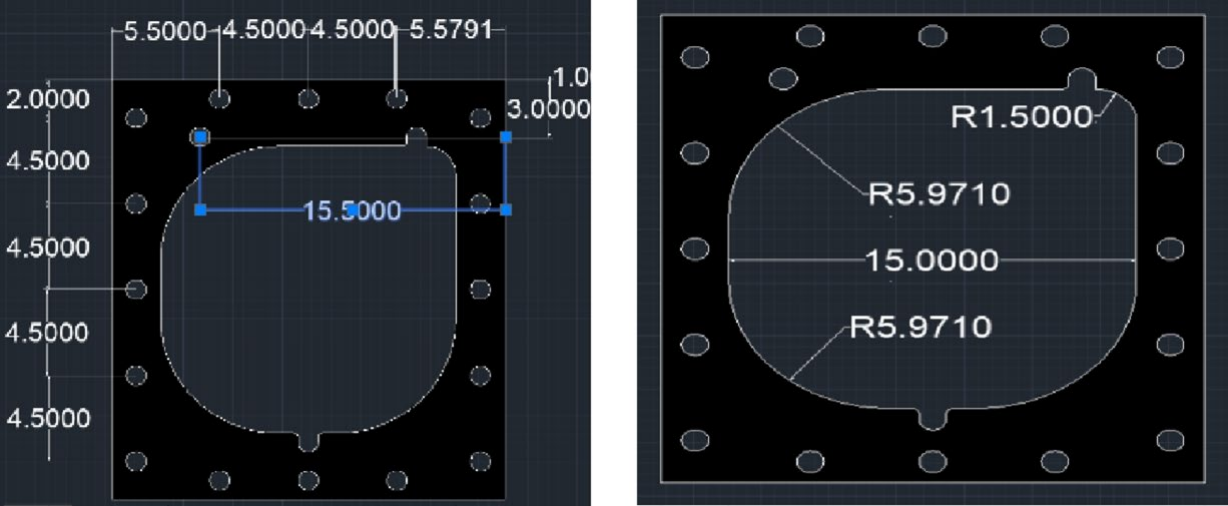
Dimensions of the gaskets.

The dimensions of gaskets as shown in Fig. 8 are selected as 200 mm X 220 mm width and height respectively. The diameter of each clamping hole is selected as 10 mm. The thickness is 6 mm. Regarding the dimension of acrylic housing plate, the dimensions of gaskets are selected as 200 mm X 240 mm width and height respectively. The diameter of each clamping hole is selected as 10 mm. The thickness is 10 mm.

The dimensions of diagram: the dimensions of gaskets are selected as 200 mm X 220 mm width and height respectively. The diameter of each clamping hole is selected as 10 mm. The thickness is 1 mm. The porosity is 120 hole/cm.

The following summarizes the key design parameters of the experimental alkaline Electrolyzer, ensuring accurate replication and efficient operation.

The final specifications of the Electrolyzer are: the Electrolyzer weight is 35 Kg. The Input power to the Electrolyzer is about 1000 W (Max). The Input Voltage for the Electrolyzer varying between 2 and 2.5 V, Changing with the change of the temperature. The aim in future is to rapidly recompensate the voltage drop happens due to the rise of the temperature, so it doesn’t affect the efficiency of the Electrolyzer Output. Maximum current absorbed by the Electrolyzer plate is 250Amps. The aim in future is to make it to 400Amps by improving some parts and the improvement of the surroundings. The max capacity for the Electrolyzer 4.5 L/min H2 (6.75 L/min HHO) (Table 1).

**Table 1:** Key design parameters of the experimental alkaline Electrolyzer.

Component	Value/specification
Electrode material	316 stainless steel (anode & cathode)
Operating voltage	2–2.6 V per cell
Current capacity	250 A (expandable to 400 A)
Gas production rate	4.5 L/min pure H_2_ (6.75 L/min HHO)
Power input	1000 W (Max)
Efficiency	~ 66% (practical), 60%-80% (theoretical)
Current density range	0.1–0.6 A/cm^2^
Dimensions (L × W × H)	20 cm × 25 cm × 40 cm
Electrode thickness	0.6 mm
Electrode spacing	10 mm
Electrolyte composition	Sodium hydroxide (NaOH) Solution
Clamping hole diameter	10 mm
Electrolyte supply tube	10 mm (outer diameter)
Gasket material	Neoprene seals
Gasket dimensions	200 mm × 220 mm, thickness: 1 mm
Structural components	Stainless steel, carbon steel, nickel alloys
Weight	Approximately 35 kg
Pressure vessel material	Stainless steel (314 Grade)
Operating temperature	50–80 °C
Control mechanism	PLC-based with sensors
Safety features	Non-return valves (NRVs), pressure relief valves

## Hydrogen-water separation system

**Fig. 9:**
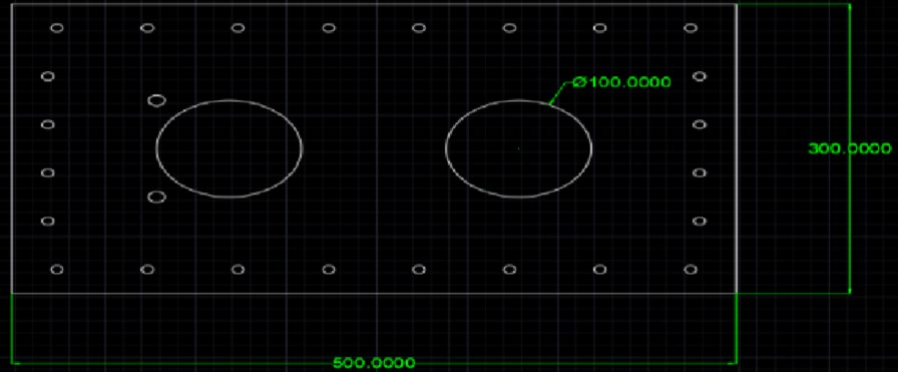
Design of hydrogen separation tank.

**Fig. 10:**
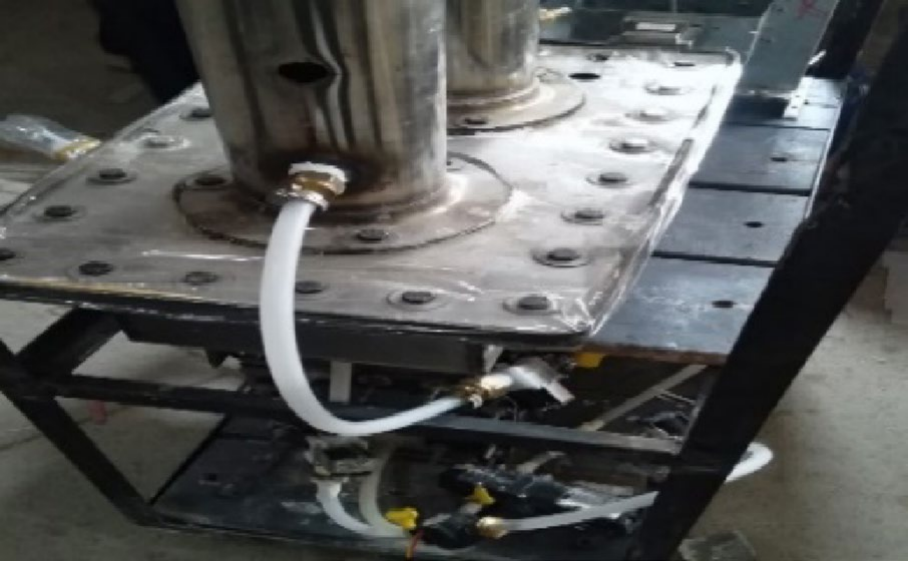
Photograph of Hydrogen separation process.

As shown in Figs. 9 and 10, the Hydrogen-Water Separation will take place in a designed tank made of stainless steel-314 which is a steel material used to resist corrosion and can withstand the harsh conditions like the raise in temperature due to mixing the Distilled water and the NAOH.

By pouring certain amount of Distilled water and certain percentage of NAOH powder salt inside the tank, the solution will undergo a certain mixing round by using an external pump, this pump will take the solution from the separation tank passing through a valve located in the bottom of the mixing tank, this valve will be opened once the tank is filled with the amount determined, the solution will pass through the valve into a water hose directed to the pump which will push this solution back to the mixing tank to ensure that the powder salt is totally diluted with the distilled water to form the electrolyte solution which will then be directed towards the Electrolyzer to start the separation reaction.

The mixing stage with the pump will take place for a whole of a 1–1.5 min to make sure that the powder salt is totally diluted and none of the salt is still saturated at the bottom of the mixing tank. Pouring the Distilled water and the Powder NAOH salt with 20% amount to maintain the concentration of the salt in the solution at 20% of the total mixture to ensure the highest efficiency output from this solution at the Electrolyzer.

By the insurances of mixing distilled water and NAOH in the mixing tank, the mixing stage stops and starts the Electrolyzer filling stage by closing the returning tank valve and opening the Electrolyzer inlet valve until the level of the capacity of the Electrolyzer and then closing the solution inlet valve and start the electrolyzing process. The electrolyzing process takes place once the inlet valve is closed and turning the power supply on the terminals of the Electrolyzers (Anode and Cathode).

The chemical equations take place in the enclosed Electrolyzer as mentioned in the previous segments producing the gases at each pole of the Electrolyzer, by directing the gases through an outlet hole at the topside of the Electrolyzer through gas hoses and a solenoid valve cutting the path of these gases for having a hand of control on these gases. Each gas return to the mixing tank at different sides of the tank through bubblers.

H_2_ gas bubbler is filled with some water at certain level so that when the H_2_ gas returns to the bubbler carrying some moisture, the water filled at the bottom of the bubbler attaches the moisture from the gas to purify it and have a better quality of a produced H_2_ gas which increases the fuel efficiency afterwards. After purifying the gas from the moisture, the H_2_ gas rises at the top of the bubbler which is closed by a Solenoid-valve to increase the flow of the gas inside the bubbler and then opening the valve to pass the gas from the bubbler to the fuel cell at certain flow or at the cylinder tank for storage at certain pressure.

## Saftey tests

The leakage test took place at different places such as:

- *Electrolyzer*:

The Electrolyzer has been filled completely and left marked for more than one day to ensure that there is no solution leakage from any point. Also, the Electrolyzer has been tested against the gas’s leakage more than one time by raising the pressure of the gases inside it and having indicators around the Electrolyzer to detect if there is any gas leakages and the test have been passed successfully.

b) *Separation Tank*.

The separation tank has been tested by being filled at its maximum capacity with water and focused under a high light intensity to see if there is any water drops coming from any point of it and the test have been passed successfully.

c) *Efficiency*.

There are a lot of factors that might affect the efficiency of production such as: Rise of the Electrolyzer temperature, the drop in the supply voltage, type and concentration of the salt inside the solution.

d) *Rise of the Electrolyzer Temperature*:

As the temperature inside the Electrolyzer increases the voltage drawn from the supply to the Electrolyzer decreases therefore, the efficiency of the Electrolyzer decreases. As the temperature decreases the power supply raises the voltage to recompensate for the drop happened in the voltage to keep the Electrolyzer at the optimum voltage value and have the highest efficiency.

e) *Supply Voltage*.

If the voltage across the Electrolyzer decreases by a small fraction it affects the efficiency of the Electrolyzer by a large value so, by taking in consideration the amount of voltage dropped from the cables and the diodes the voltage that needs to be across the Electrolyzer needs to be maintained at the optimum voltage value, even if the surroundings across the Electrolyzer changes the supply needs to rapidly adapt with it so the efficiency doesn’t be affected.

f) *Type and concentration of salt*.

As mentioned in the segments before the type of the powder salt is very significant in the process of electrolyzing the electrolyte cause every type of salt has a different specific conductivity and therefore, it affects on the concentration of this salt which be considered in the solution which will affect on the efficiency of the production after all.

g) *Mixing and external Pumps*.

Pump located between the separation tank and the Electrolyzer, responsible for mixing the solution in a closed loop through the separation tank and then force the solution after ensuring that the salt is completely diluted and none of it still saturated at the bottom of the tank and used to push the solution towards the Electrolyzer to start the electrolyzing process. This pump is responsible for filling the distilled water into the separation tank to start the mixing process with the powder salt. This pump configured before the separation tank.

## Working principle and operational sequence of the electrolayser

**Fig. 11:**
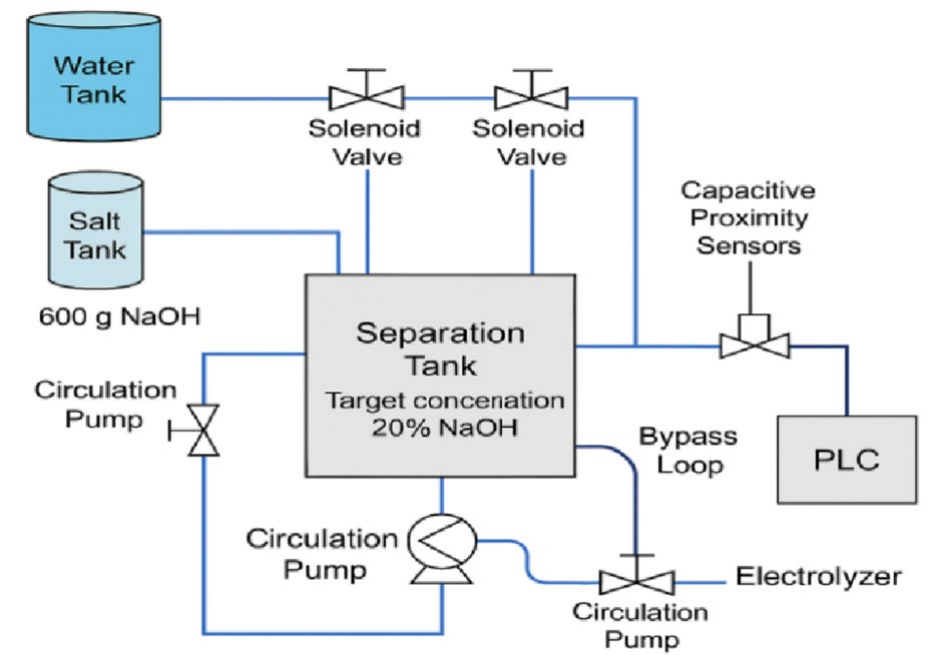
Separation tank operational sequence.

The separation tank follows a systematic, multi-step operational logic presented in Fig. 11. Step 1: Mixing — Water and salt are introduced and combined using an external pump. Step 2: Electrolyzer Charging — Once mixing is completed, solution is injected into the Electrolyzer. Step 3: Gas Collection - As the electrolysis proceeds, H_2_ and O_2_ gasses are created and steered back to the tank. Step 4: Bubbling and Purification - Each gas enters its assigned bubbler where moisture is eliminated. Step 5: Gas Outlet — Purified hydrogen is routed to storage tanks or fuel cells, while oxygen is either vented or reused. The Electrolyzer operating temperature is 80 °C at 10 bar pressure, the NaOH concentration is 20%.

## Instrumentation and measurement system

**Fig. 12:**
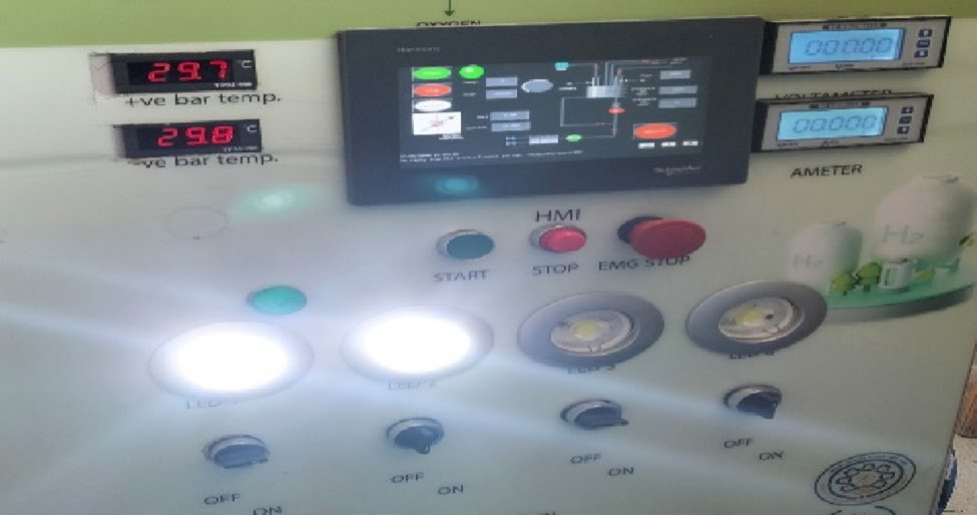
Monitored sensors on HMI.

Real-time instrumentation plays a major role in the green hydrogen pilot system’s performance, safety, and automation capabilities. Pressure, flow, temperature, level, and electrical parameters may all be continuously monitored and intelligently controlled thanks to the integration of a wide range of sensors and measurement instruments. The main tools used in the setup are described in this section as illustrated in Fig. 12.

Stainless steel is used for the proposed Electrolyzer. The material was chosen because it provides a practical balance between corrosion resistance, mechanical strength, availability, manufacturability, and cost, making it well suited for a pilot-scale alkaline water electrolysis system. In addition, 316 stainless steel exhibits good chemical stability in alkaline electrolytes and has been widely reported in the literature for laboratory and pilot-scale alkaline Electrolyzers.

A) *Pressure Monitoring*.

The system’s hydrogen and oxygen gas pressures are continuously monitored by two pressure transmitters. For real-time safety inspections, these devices are connected to the PLC and positioned strategically near the gas bubblers’ output. Measurement range: 0–10 bar; analog output signal: 4–20 mA. It is made of a 316 L stainless steel diaphragm that can withstand pressured and corrosive conditions. The purpose is to initiate automated relief operations and pressure alarms in the event of anomalous circumstances.

b) *Hydrogen Flow Measurement*.

This mass flow meter delivers reliable, thermal-based flow measurements of hydrogen gas, vital for process efficiency and validation. Technology: Constant Temperature Anemometry (CTA). Display: OLED for instantaneous readouts. Output: Analog signal + Modbus/RS-485 digital communication. Functions: Flow control, gas selection, and alarm activation. The hydrogen volume is measured by flow meter as in Fig. 15 at 80 °C, 10 bar pressure and 20% NaOH concentration.

c) *Electrolyte Circulation and Filling*.

This flow sensor is positioned in the electrolyte circulation line to certify the appropriate working of the mixing and filling cycles. Function: Monitors water flow during NaOH solution preparation. Sends real-time flow rate data to the PLC to guarantee accurate electrolyte delivery. Ensures pump operation and prevents dry-run circumstances.

*d) Electrolyte Level Detection*.

To ensure accurate electrolyte levels during mixing and operation, capacitive sensors are installed at various heights in the separation tank and Electrolyzer inlet. The purpose is to prevent overfilling, automate the switching between mixing, filling, and shutdown cycles and withstand corrosive environments using acrylic protective coating.

e) *Temperature Monitoring*.

Temperature sensors are directly mounted on the anode and cathode terminals of the Electrolyzer to approximate internal electrolyte temperature. These readings ensure safe operation within thermal thresholds. The purpose is to prevent thermal degradation of materials, trigger system shutdown in the event of overheating, enable temperature-dependent voltage/current adjustments.

f) *Electrical Monitoring*.

Voltage and Current Sensors are used to monitor the real-time voltage applied to the Electrolyzer stack. Range: Configured per system requirements. Interface: Panel-mount with digital display. DC Ammeter measures Electrolyzer current using external shunt resistance.RS-485 Modbus-enabled for integration with PLC. Dual programmable alarm relays for current safety threshold violations. The PLC used in this work is Schneider PLC (Model M200C40T), the PLC speed for high-speed inputs of 4 channels is up to 100 KHZ, the internal memory is 512 Bytes internal Flash and external memory is Supports MicroSD card up to 32 GB for program storage and duplication, Program execution time is 0.2 µs per Boolean instruction.

g) *Fuel Cell Sensors*.

The hydrogen fuel cell section is equipped with its own sensing array to monitor Input gas pressure and purity, Temperature of the stack and Electrical output (voltage/current**).** These sensors ensure optimal power conversion performance and allow for predictive maintenance and efficiency optimization.

**Fig. 13:**
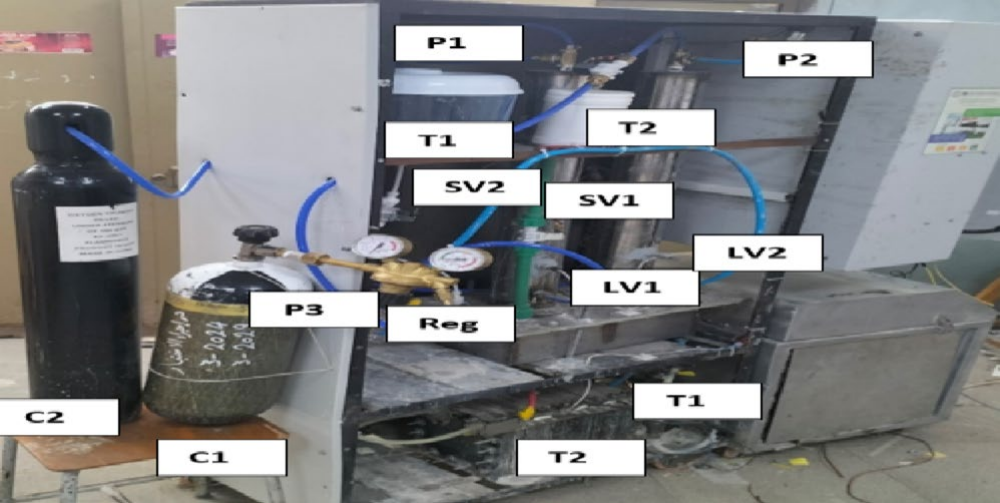
Installed sensors including pressure, temperature, and level sensors on the physical system.

**Fig. 14:**
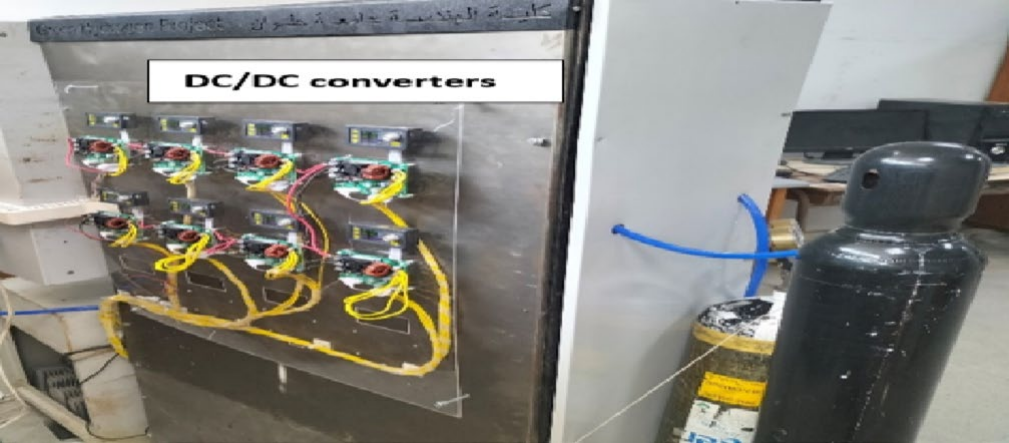
Custom-built DC/DC converter cabinet.

**Fig. 15:**
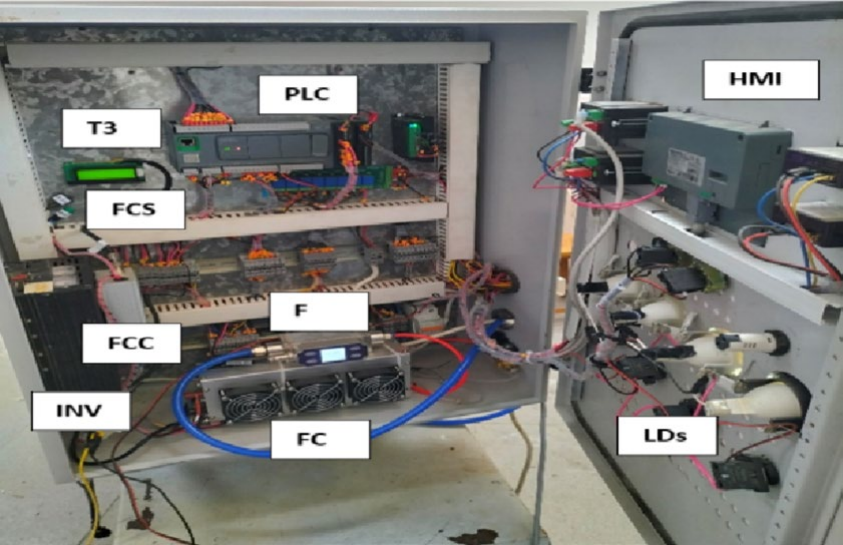
Real sensor including Gas Flow sensor wiring, PLC connections, and HMI interface.

The spatial distribution of the sensors across the pilot system is illustrated in Figs. 2 and 3. A real photograph of the setup with sensor placements is shown in Figs. 13, 14 and 15, captures actual sensor readings displayed on the HMI screen. Table 2 shows the description of sensor labels and their functional roles in the pilot system.

**Table 2:** Description of sensor labels and their functional roles in the pilot system.

Label	Component	Function
P1	Hydrogen Pressure Transmitter	Monitors gas pressure post-Electrolyzer
P2	Oxygen Pressure Transmitter	Monitors oxygen bubbler pressure
P3	Electrolyte Pressure Monitor	Safety feedback for tank pressure
T1/T2	Electrolyzer Temperature Sensors	Monitors temperature at cathode/anode busbars
T3	Fuel Cell Temperature	Ensures safe thermal environment for Fuel Cell
C1/C2	Capacitive Level Sensors	Detects electrolyte levels in bubblers
FC	Fuel Cell Unit	Converts stored H_2_ to electricity
FCC	Fuel Cell Controller	Manages load control and sensor interfacing
F	Hydrogen Flow Sensor	Measures hydrogen production rate post-separation
FCS	Fuel Cell switches	Control the Fuel cell starting and stopping actions
LDs	AC /DC load Lamps	Show AC and DC supplied from Fuel Cell
INV	Inverter	Converts DC output from fuel cell to AC for load supply
PLC	Programmable Logic Controller	Main automation unit with all sensor and actuator logic
HMI	Human-Machine Interface	Displays all sensor readings and allows operator interaction

## Control architecture overview

**Fig. 16:**
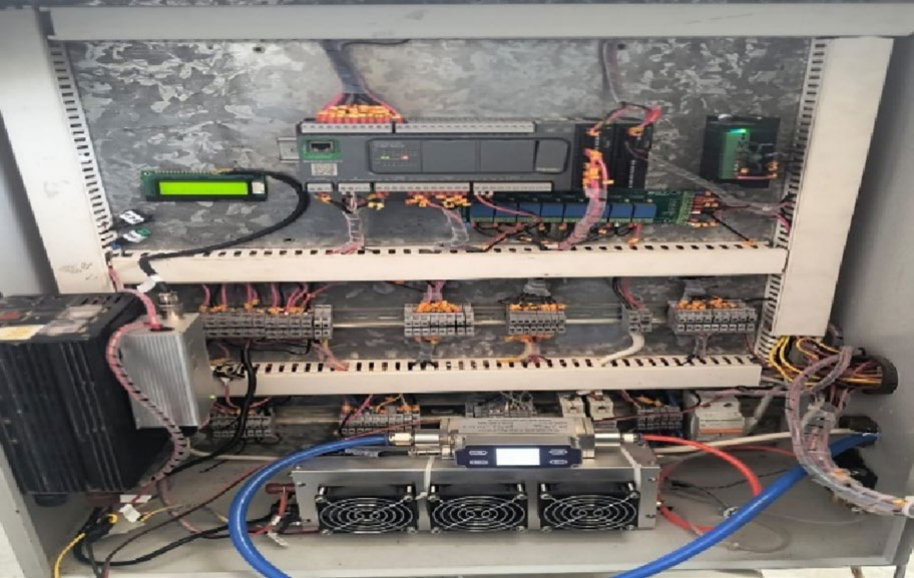
Internal layout of the control panel featuring the Schneider PLC.

The control architecture is built around a Schneider PLC (Model M200C40T) equipped with both digital and analog I/O modules, supported by a HMI touch panel (Model GXU3512) for real-time operation and monitoring shown in Fig. 12. The PLC executes sequential and conditional logic to manage sensors, actuators, pumps, power regulation, and gas routing valves. Communication Protocol: Modbus RTU over RS-485. Programming Environment: Schneider So Machine / EcoStruxure Machine Expert. Main Control Modes shown in Fig. 16 shows the electrolyte preparation, Electrolyzer filling and activation, gas monitoring and purification, system alarms and fail-safe actions.

### PLC control sequences

The PLC program is designed with modular, event-driven logic to ensure precise operation of all phases:

i. *Mixing Phase*:

Solenoid valves open to admit distilled water and NaOH salt into the mixing tank. A circulation pump activates to homogenize the solution for 10–15 s. Level sensors monitor fill height; system locks if levels are not within range.

ii. *Filling Phase*:

Electrolyzer inlet valve opens for 110 s to charge the cell with the prepared electrolyte. The pump deactivates, and all valves are closed before initiating electrolysis.

iii. *Electrolysis Phase*:

Power supply is enabled via relay control and the variac is driven using a TB6600 stepper motor driver based on PLC-generated Pulse Train Output (PTO).Voltage and current are continuously monitored. Alarms are triggered for overcurrent or undervoltage conditions.

### Gas monitoring and delivery sequnce

At first, H_2_ and O_2_ gases return through check valves into the separation tank bubblers then, pressure sensors monitor gas build-up. The solenoid valves open to either vent or route gases to storage/fuel cells depending on HMI commands.

For Safety and Fault Handling, Overpressure detection triggers gas venting.Over-temperature reading disables the power supply.Flow obstruction or low electrolyte level halts the mixing process and issues HMI alerts.

**Fig. 17:**
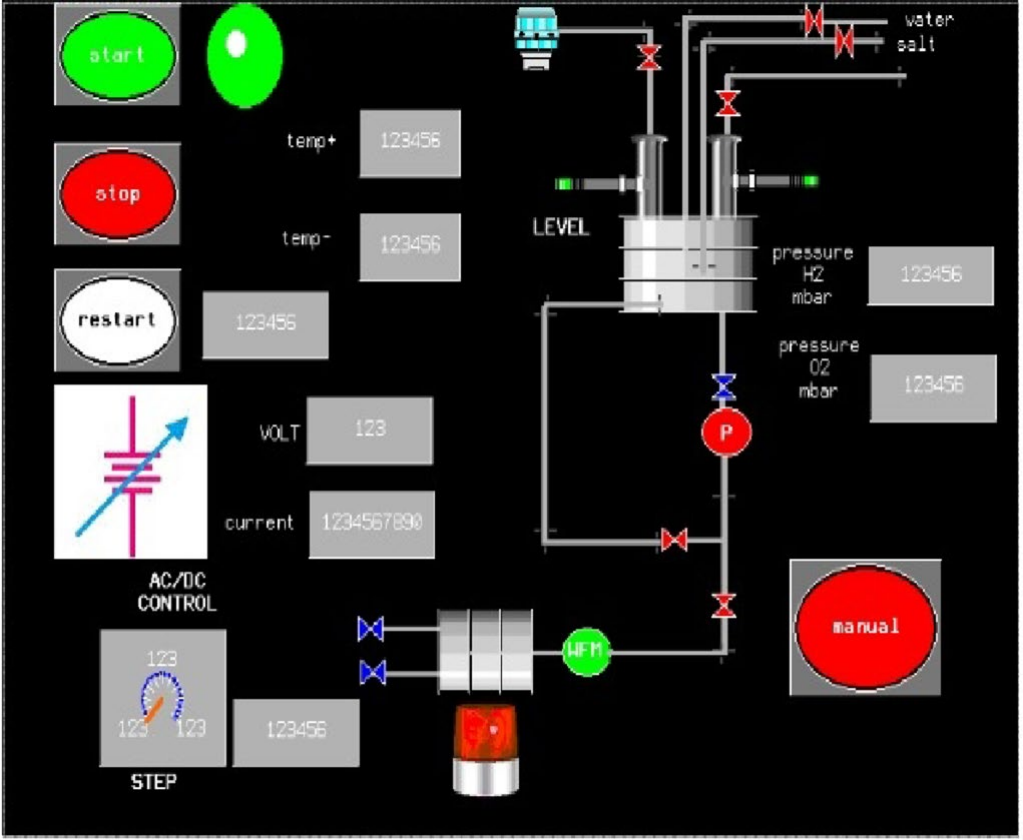
HMI Interface Functions.

The HMI (Model GXU3512) as show in Fig. 17 provides start/stop/emergency stop controls, live monitoring for voltage, current, pressure, flow, temperature, and level. Manual override for independent control of pumps, solenoid valves, and power supply. Alarms & status indicators for visual/audio alerts for faults and system readiness and running status. Data logging capability for real-time value display for on-site diagnostics Possible export interface for future cloud or SCADA integration.

## Operational results and data analysis

The main performance outcomes from the Green Hydrogen Pilot System’s operation are shown in this section. The results are based on several experimental trials carried out with different loads, operating modes (grid-connected and PV-powered), and ambient circumstances. Integrated sensors and the PLC logging interface were used to gather data, which was subsequently verified using laboratory-grade equipment and manual measurements.

### Hydrogen production performance

Under regulated circumstances, the system showed stable hydrogen generation. The practical pilot system efficiency was subsequently calculated as the ratio of the chemical energy stored in the produced hydrogen to the electrical energy supplied to the Electrolyzer. In addition, the relevant equations, assumptions, and calculation procedure to improve the transparency and reproducibility of the reported efficiency values (62–66%)^8^.

1 $${\eta _{{\mathrm{el}}}}=\frac{{{{\dot {m}}_{{{\mathrm{H}}_2}}} \times HH{V_{{{\mathrm{H}}_2}}}}}{{{P_{{\mathrm{el}}}}}}$$

where: $\:{\eta\:}_{\mathrm{el}}$ = Electrolyzer efficiency; $\:{\dot{m}}_{{\mathrm{H}}_{2}}$= hydrogen production rate (kg/s); $\:HH{V}_{{\mathrm{H}}_{2}}$ = higher heating value of hydrogen (≈ 39.4 kWh/kg or 141.8 MJ/kg); $\:{P}_{\mathrm{el}}$ = electrical power input (kW).

Table 2 shows the practical data of the Electrolyzer. As the electrolyte temperature rose, the production rate was constant for up to 45 min, with only minor drops in gas generation. It is anticipated that improved heat management would boost performance even more.

**Table 3:** Practical data of the Electrolyzer.

Electrolyzer input voltage	2.4–2.6 V DC
Electrolyzer input current	210–250 A
Electrolyzer input power	500–700 W
Hydrogen output rate	Peak: 4.5 L/min of pure H_2_ Equivalent: 6.75 L/min of HHO mixture
Energy conversion efficiency Theoretical (Thermoneutral)	~ 67%
Energy conversion efficiency (practical)	~ 62–66%, based on gas volume and input energy

### Electrolyzer voltage-current characteristics

As indicated in Table 3, a number of measurements are made to evaluate the Electrolyzer’s V-I behavior. The cell functions in the ohmic region of its characteristic curve, according to the results, with rising voltage being almost linear to current density.

**Table 4:** V-I characteristics of the electrolyzer.

Current (A)	Current density (A/cm^2^)	Voltage (V)
88	0.2	2.3
154	0.35	2.425
220	0.5	2.5
250	0.57	2.6

This reaction validates the 20% NaOH electrolyte’s effective ionic conductivity and balanced internal resistance.

### Electrolyte behavior and thermal response

The operating range is 50–58 °C (without active cooling), and the starting electrolyte temperature is 26–28 °C. No sediment was seen, and the recirculation loop and mixing accuracy guaranteed a consistent dilution of NaOH. Values reported by DCV-10 S and DCA-10 S meters were within ± 2% of external instruments. Under 6 bar, pressure sensors successfully continued to function. Tank condition was consistently reported by capacitive level sensors, guaranteeing safe filling and electrolyte reuse. The PLC responded to sensor input and trigger conditions in less than 250 milliseconds, guaranteeing reliable system behavior and secure operation.

The consistency and dependability of operations Consistency from run to run System stability and repeatable gas output were obtained after more than ten trials. Throughout every cycle, neither the Electrolyzer nor the separation tank had any leaks. The HMI interface allowed for consistent and user-friendly automated startup and shutdown procedures.The performance of hydrogen systems with grid and solar power supplies is compared in Table 4.

**Table 5:** Mode comparison: grid vs. PV power.

Parameter	Grid-powered mode	PV-powered mode
Power supply stability	Highly stable	Dependent on sunlight intensity
Electrolyzer activation	Immediate	Delayed during cloudy conditions
HMI/PLC operation	Full functionality	Operates via battery backup
Practical feasibility	Ideal for testing	Sustainable, scalable deployment

PV operation verified the system’s potential for off-grid and renewable-based hydrogen production, especially in remote or solar-rich areas, even though grid power offered perfect conditions for laboratory validation (Table 5).

### Safety and efficiency tests conducted

Throughout the development phase, ensuring the Green Hydrogen Pilot System operated safely and effectively was a top priority. To verify the system’s mechanical integrity, thermal stability, control response, and gas purity, a thorough set of tests was carried out (Table 6). These tests were crucial for improving the automation and protection logic of the system in addition to confirming design assumptions. Table 4 displays the summaries of the tests.

**Table 6:** Summary of safety and efficiency tests.

Test	Objective	Method	Result/outcome
Electrolyzer leak test	Confirm seal integrity under operating conditions	Filled with 20% NaOH; monitored for 24 h; visual and bubble testing	No leaks detected; Electrolyzer validated for safe operation
Separation tank leak test	Ensure weld and joint durability	Pressurized water test; high-intensity lighting inspection	No leakage observed; structural integrity confirmed
Gas pressure safety test	Verify overpressure control and venting mechanism	ETRANS-P01 sensors; PLC-triggered alarms; automated relief valve activation	Successful venting at threshold; alarm-response sequence validated
Moisture removal test	Assess hydrogen gas purity after bubbling and filtering	Dual-stage filtration: bubbler + silica gel + pneumatic filter (AF4000-04)	Hydrogen output confirmed dry; no condensation in fuel cell line
Temperature control test	Monitor and regulate electrolyte temperature	ZFX-900 digital sensors on electrodes; automated cutoff at 60 °C	Overheat response triggered as designed; safe temperature maintained
Electrical monitoring accuracy	Validate DCV-10 S & DCA-10 S against calibrated instruments	Load variation testing; multimeter comparison; alarm simulation	All readings within ± 2% accuracy; alarms triggered appropriately
PLC functional logic test	Confirm proper execution of automation sequences and safety interlocks	Simulation of faults (flow obstruction, pressure spikes, sensor failures)	All fault scenarios handled; process halted or rerouted per design logic
Electrolyte mixing consistency	Ensure proper NaOH solution concentration	Controlled pump loop; timed recirculation; NaOH: water ratio monitored	Stable mixing achieved; 20% concentration maintained across trials
Non-return valve functionality	Prevent backflow of gases and electrolyte	Installed on gas and fluid lines; visual verification during operation	Flow remained unidirectional; no contamination detected
Voltage stability	Maintain proper Electrolyzer voltage during fluctuating loads	Custom DC power setup; monitored via DCV-10 S; PLC-controlled variac	Voltage held within target range (2.4–2.6 V); minor variations auto-corrected

## Comparison and discussion

While industrial systems usually rely on expensive PLC/SCADA designs, the majority of laboratory-scale Electrolyzers use microcontroller-based or manual control tactics. By combining a Schneider M200 PLC with an HMI interface, the suggested solution fills this gap by offering real-time monitoring, automated operation, data collecting, and safety protection on a small and affordable platform. The suggested method prioritizes automation, operational dependability, and ease of industrial deployment in contrast to traditional laboratory prototypes that are mainly concerned with hydrogen production performance. In order to enable stable system operation and minimize operator intervention, the designed control architecture continuously monitors voltage, current, temperature, water level, and hydrogen flow rate. When compared to industrial hydrogen production systems, the use of commercially available components such as the Schneider M200 PLC, common industrial sensors, and a low-power Electrolyzer stack (0.5 kW) significantly lowers implementation costs while preserving high operational flexibility. Furthermore, by adding more Electrolyzer stacks without significantly altering the control circuitry, the modular architecture allows for future growth. The current system’s control philosophy can easily be expanded to bigger Electrolyzer facilities coupled with renewable energy sources and smart microgrids, even though it is designed for laboratory and pilot-scale hydrogen generation. As a result, the suggested platform offers a workable middle ground between industrial hydrogen generating systems and research-scale prototypes, balancing automation capability, scalability, implementation cost, and operational safety.

## Conclusions and future directions

Since many electrical energy storage systems are often polluting to the environment and expensive, this research proposed storing electrical energy in a green hydrogen form resulting from storing energy from solar panels. In this research, the precise design of the water analyzer was carried out in all its stages, and with a detailed explanation of the design process, the oxygen and hydrogen storage tanks were designed, the hydrogen separation process was carried out. The green hydrogen production and storage process was fully controlled using a PLC device, and the operating voltage of the water analyzer, the operating current, and the safety processes related to hydrogen production and storage were maintained. Many of the necessary sensors were installed to maintain the production and storage process for temperature sensors, pressure sensors, current sensors, and hydrogen gas flow rate sensors. The developed 0.5 kW alkaline Electrolyzer represents a pilot-scale platform that bridges laboratory research and larger hydrogen production systems. Although its hydrogen production capacity is modest, the system is fully scalable because larger installations can be realized by increasing the electrode active area, stacking additional cells, or integrating multiple electrolyzer modules operating in parallel. This modular approach facilitates capacity expansion while maintaining similar control strategies and operating conditions. From an economic perspective, the 0.5 kW system offers a cost-effective solution for prototype validation, control algorithm development, educational purposes, and small-scale renewable energy applications, with significantly lower capital investment than industrial-scale electrolyzers. Furthermore, the PLC/HMI-based control architecture employed in this work is readily transferable to higher-power systems, requiring only minor modifications to accommodate increased sensor capacity and power electronics. Consequently, the proposed system provides a practical foundation for future scale-up toward kilowatt- and megawatt-scale hydrogen production, where economies of scale are expected to reduce the specific hydrogen production cost while improving overall system efficiency and commercial viability.

The future directions inthis area are:

- Building on the success of the pilot implementation, several avenues are proposed for further enhancement and scaling for Hydrogen Compression and Storage Integration.
- Optimization of Smart Control and Renewable Energy completely automates the scheduling of hydrogen production, Electrolyzer load, and PV input. Combine intelligent battery-buffering logic with MPPT (maximum power point tracking) and using AI algorithms for energy forecasting, predictive maintenance, and dynamic process optimization.
- Increased Data Analysis and Instrumentation.
- For increased safety and compliance, include hydrogen detectors, humidity probes, and gas purity monitors. IoT gateways can be used to enable cloud-based data visualization and remote monitoring. In adtion to uncertainty analysis.
- Scaling for industrial applications. regarding scaling for use in industrial settings design multi-stack Electrolyzer systems using the pilot’s lessons learnt. collaborate with regional businesses to develop an affordable, modular green hydrogen platform specifically designed for Egypt’s water and energy industries.

## Data Availability

All data generated or analyzed during this study are included in this published article.
